# Delivery of chemotherapeutic agents using drug-loaded irradiated tumor cells to treat murine ovarian tumors

**DOI:** 10.1186/1423-0127-17-61

**Published:** 2010-07-26

**Authors:** Daejin Kim, Talia Hoory, Archana Monie, Annie Wu, Wei-Ting Hsueh, Sara I Pai, Chien-Fu Hung

**Affiliations:** 1 Department of Pathology, Johns Hopkins Medical Institutions, Baltimore, Maryland, USA; 2 Department of Oncology, Johns Hopkins Medical Institutions, Baltimore, Maryland, USA; 3 Department of Otolaryngology and Head & Neck Surgery, Johns Hopkins Medical Institutions, Baltimore, Maryland, USA; 4 Department of Anatomy, Chung-Ang University College of Medicine, Dongjak-Gu, Seoul, South Korea; 5 Department of Radiation Oncology, National Cheng Kung University Hospital, Taiwan

## Abstract

**Background:**

Ovarian cancer is the leading cause of death among women with gynecologic malignancies in the United States. Advanced ovarian cancers are difficult to cure with the current available chemotherapy, which has many associated systemic side effects. Doxorubicin is one such chemotherapeutic agent that can cause cardiotoxicity. Novel methods of delivering chemotherapy without significant side effects are therefore of critical need.

**Methods:**

In the current study, we generated an irradiated tumor cell-based drug delivery system which uses irradiated tumor cells loaded with the chemotherapeutic drug, doxorubicin.

**Results:**

We showed that incubation of murine ovarian cancer cells (MOSEC) with doxorubicin led to the intracellular uptake of the drug (MOSEC-dox cells) and the eventual death of the tumor cell. We then showed that doxorubicin loaded MOSEC-dox cells were able to deliver doxorubicin to MOSEC cells in vivo. Further characterization of the doxorubicin transfer revealed the involvement of cell contact. The irradiated form of the MOSEC-dox cells were capable of treating luciferase-expressing MOSEC tumor cells (MOSEC/luc) in C57BL/6 mice as well as in athymic nude mice resulting in improved survival compared to the non drug-loaded irradiated MOSEC cells. Furthermore, we showed that irradiated MOSEC-dox cells was more effective compared to an equivalent dose of doxorubicin in treating MOSEC/luc tumor-bearing mice.

**Conclusions:**

Thus, the employment of drug-loaded irradiated tumor cells represents a potentially innovative approach for the delivery of chemotherapeutic drugs for the control of ovarian tumors.

## Introduction

Ovarian cancer is the leading cause of death among women with gynecologic malignancies and is the eighth most common cancer in the United States [1,2]. Most patients who are diagnosed with ovarian cancer are detected at an advanced stage (III/IV), often presenting with complications associated with intraperitoneal metastasis. Unfortunately, less than half of the women diagnosed with ovarian cancer survive 5 year post-diagnosis [1,3]. Current chemotherapies are useful in the control of advanced stages of ovarian cancer but have many toxic side effects [4-6]. Thus, there is a critical need for alternative approaches to administer chemotherapeutic agents to control advanced stages of ovarian cancer without serious side effects.

Doxorubicin, which is part of the anthracyline family, has been successfully applied to treat a variety of tumors including ovarian cancer (for review see [7]). While doxorubicin is more effective than its structural precursor, daunorubicin, the major side effects of the drugs are similar. Studies have shown that the toxicity of doxorubicin can lead to chronic cardiomyopathy [8-10]. Thus, some attempts have been made to diminish the toxicity of doxorubicin. One currently administered form of doxorubicin is DOXIL^®^, whereby doxorubicin is encapsulated by lipids to prolong the circulation of the drug in the bloodstream [11]. Although the liposome protects some cells from doxorubicin, they can reach systemic circulation and the drug can still reach heart tissue to cause damage.

In the current study, we hypothesized that local administration of doxorubicin delivered by irradiated tumor cells may reduce the dose required to treat murine ovarian cancer cells and decrease the systemic circulation of doxorubicin. We showed that preparation of murine ovarian cancer cells (MOSEC) with doxorubicin led to the intracellular uptake of the drug (MOSEC-dox cells). We then showed that doxorubicin loaded MOSEC-dox cells were able to deliver doxorubicin to MOSEC cells in vivo. Thus, local delivery of chemotherapeutic drugs by tumor may represent a potentially innovative approach for the control of ovarian tumors.

## Materials and methods

### Mice

Female C57BL/6 and athymic nude mice (6-8 wks) were acquired from the National Cancer Institute (Frederick, MD). All animals were maintained under specific pathogen-free conditions, and all procedures were done according to approved protocols and in accordance with recommendations for the proper use and care of laboratory animals.

### Cell lines and reagents

The HPV-16 E7-expressing murine tumor model, TC-1, has been described previously [12]. In brief, HPV-16 E6, E7, and the *ras *oncogene were used to transform primary C57BL/6 mice lung epithelial cells to generate the TC-1 cell line. The MOSEC cell line was generated as described previously [13]. The MOSEC cell line was originally derived from murine ovarian surface epithelial cells [13]. MOSEC-luciferase (MOSEC/luc) cells were generated as described previously [14]. MOSEC cells were transduced with a retrovirus containing luciferase. In order to generate a retrovirus containing luciferase, a pLuci-thy1.1 construct expressing both luciferase and thy1.1 was made. Firefly luciferase was amplified by PCR from pGL3-basic (Promega) using the 5' primer CGGAGATC TATGGAAGACGCCAAAAAC and the 3' primer CGGGTTAACTTACACGGCGATCTTTCC. The amplified luciferase cDNA was inserted into the *Bgl*II and *Hpa*I sites of the bicistronic vector pMIG-thy1.1. Both luciferase and thy1.1 cDNA are under the control of a single promoter element and separated by an internal ribosomal entry site (IRES). The pLuci-thy1.1 was transfected into Phoenix packaging cell line and the virion-containing supernatant was collected 48 h after transfection. The supernatant was immediately treated using a 0.45-mm cellulose acetate syringe filter (Nalgene, Rochester, NY, USA) and used to infect MOSEC cells in the presence of 8 mg/ml Polybrene (Sigma, St Louis, MO, USA). MOSEC/luc cells were sorted using preparative flow cytometry of stained cells with Thy1.1 antibody (BD, Franklin Lakes, NJ, USA). MOSEC-GFP cells were generated with a GFP-expressing lentivirus. Briefly, the lentiviral vector pCDH1-EF1-GFP was transfected into a Phoenix packaging cell line using lipofectamine (Invitrogen, Carlsbad, CA, USA) and the virion-containing supernatant was collected 48 hours after transfection. The supernatant was then filtered through a 0.45 mm cellulose acetate syringe filter (Nalgene, Rochester, NY, USA) and used to infect MOSEC cells in the presence of 8 mg/ml Polybrene (Sigma-Aldrich, St Louis, MO, USA). Transduced cells were isolated using preparative flow cytometry with GFP signal. The growth rate of all transduced cell lines was comparable with those of the parental, non-transduced cell lines (data not shown). Doxorubicin-HCL (D1515, Sigma-Aldrich, St Louis, MO, USA) was reconstituted with 0.9% NaCl normal saline and kept at 4°C for up to three weeks.

### Determination of drug concentration inside doxorubicin-treated cells

MOSEC cells (1 × 10^6^/ml) were cultured with complete media in the presence of different concentrations of doxorubicin (specifically 1, 10, 50, 100 μg/ml) for 2 hours at 37°C. The cells were then centrifuged at 10,000 rpm for 2 mins and the supernatant aspirated. The intracellular drug concentration was then determined within the remaining cell pellets. The cell pellets were lysed with protein extraction buffer (Pierce, Rockford, IL) and a 1:1 volume of DMSO was added. The concentration of drug was determined using a spectrophotometer at a 470 nm wavelength. Standard solutions of doxorubicin were made with media or extraction buffer with DMSO and used to generate a standard curve. Linear regression analysis was performed to generate the regression equation: y = 0.1607x -0.2143 with R^2 ^= 0.9102.

### Drug uptake, viability and proliferation of cells

MOSEC and MOSEC/luc cells (1 × 10^6^/ml) were cultured in the presence of indicated doses of doxorubicin (specifically, 0.01, 0.1, 1, 10, 50, 100 μg/ml) at 37°C for 2 hours. Analysis was performed on a BD FACScan with CellQuest software (BD Biosciences Immunocytometry Systems, Mountain View, CA). After 2 hours of incubation with the drug, 5 × 10^4 ^cells/well of doxorubicin-treated MOSEC/luc cells were placed into 96-well plates with complete medium. D-Luciferin (potassium salt; Xenogen/Caliper Life Sciences, Alameda, CA) at a concentration of 150 *μ*g/ml was added to each well 7-8 minutes before imaging at 24 hrs. The imaging time was 30 seconds/plate. A MTT assay was performed with doxorubicin-treated MOSEC cells at 24 hours. The cells were then divided into 96-well plates. The MTT solution (30 μl of a 5 mg/ml solution) was added to the drug treated cancer cells and incubated for 4 hours. 100 μl DMSO was added to dissolve formazan crystals under vigorous shaking for 30 minutes which was followed by detection of absorption at OD 570 nm using a microplate reader (Molecular Probes, Invitrogen, Eugene, OR).

### Drug transfer *in vitro *and *in vivo*

MOSEC cells pre-treated with doxorubicin (100 μg/ml) were mixed with MOSEC-GFP cells (5 × 10^5^/well in 24-well plates) according to the indicated ratios (5 × 10^3 ^(100:1), 1 × 10^4 ^(50:1), 2 × 10^4 ^(25:1), or 5 × 10^4 ^(10:1)/well). After 24 hours, all the cells were collected and analyzed by flow cytometry. In order to confirm the necessity of cell to cell contact in the transfer of the drug, MOSEC-GFP cells (5 × 10^5^/well in 24-well plates) were cultured in the bottom well of transwell plates (Corning Costar, Acton, MA) and MOSEC cells (5 × 10^4^/well in 24-well plates) pre-treated with doxorubicin (100 μg/ml) were cultured in the upper chamber. After 24 hours, all of the cells in the bottom well were collected and analyzed by flow cytometry. For detecting transfer of drug *in vivo*, female C57BL/6 mice were inoculated with MOSEC-GFP (1 × 10^6^/mouse) via the intraperitoneal route. After 24 hours, 2 × 10^4 ^(50:1) or 1 × 10^5 ^(10:1) MOSEC cells pre-treated with doxorubicin (100 μg/ml, 2 hrs) were injected into MOSEC-GFP tumor bearing mice. MOSEC-GFP cells or MOSEC-dox cells alone (1 × 10^6 ^/mouse) were injected into mice as controls. 24 hours after injecting the drug treated cells, mice from all groups were sacrificed with CO_2 _inhalation. Sterile PBS (10 ml) was injected into the peritoneum of each mouse to obtain peritoneal cells. Peritoneal cells (1 × 10^6 ^/mouse) were then analyzed by flow cytometry.

### Characterization of tumor cell death by drug-treated tumor cells *in vitro*

MOSEC cells treated with a high dose (100 ug/ml) of doxorubicin were co-cultured with MOSEC/luc cells (5 × 10^4^/well in 24-well plates) at different ratios (5 × 10^2^(100:1), 1 × 10^3 ^(50:1), 2 × 10^3 ^(25:1), or 5 × 10^3 ^(10:1)/well). D-Luciferin (150 μg/ml) was added at different time points (just after mixing, on day1, and on day 2) and incubated for 7-8 min. An integration time of 30 seconds was used for luminescence image acquisition. Data was obtained on day 2.

### Characterization of anti-tumor effects by drug-loaded tumor cells in C57BL/6 mice

Naïve female C57BL/6 mice were inoculated intraperitoneally with 5 × 10^5 ^live MOSEC/luc cells per mouse. On day 4 after tumor inoculation, tumor bearing mice were injected with low (2 × 10^5^/mouse) or high (2 × 10^6^/mouse) numbers of drug-treated, irradiated MOSEC cells. Tumor-bearing mice were also injected with 2 × 10^5 ^irradiated MOSEC cells as a control. For drug-treated, irradiated tumor cells, MOSEC cells were incubated for 2 hours with 100 μg/ml of doxorubicin and then subjected to 100,000 cGy/min for 10 minutes. Tumor growth was assessed with luminescence image acquisition on day 0 after treatment with drug treated cells and, subsequently, on a weekly basis. The mice were injected with 0.2 ml of 15 mg/ml D-luciferin. Detection of luminescence activity indicating relative tumor development was then performed using a Xenogen IVIS 200 Imaging System.

### Characterization of anti-tumor effects of drug-loaded tumor cells in nude mice

Athymic nude mice (B6 background) were inoculated intraperitoneally with 2.5 × 10^5 ^live MOSEC/luc cells per mouse. On day 4, tumor bearing mice from each group (5mice/group) were treated with irradiated MOSEC cells (2 × 10^6^/mouse) treated either with low (10 μg/ml) or high (100 μg/ml) doses of doxorubicin. Tumor growth was monitored on a weekly basis from the day of MOSEC/luc tumor challenge using the bioluminescence imaging method mentioned above.

### Comparison of the different treatment regimens

The concentration of drug inside the doxorubicin-treated MOSEC cells was determined as described. Naïve female C57BL/6 mice were challenged intaperitoneally with 5 × 10^5 ^live MOSEC/luc cells per mouse. On day 4, tumor bearing mice were injected with 0.5 mg/kg (10 μg/mouse) of doxorubicin. To compare the effects of treatment on tumors, drug-loaded irradiated MOSEC cells (2 × 10^6^/mouse, 100 μg/ml for 2 hrs) were injected into tumor-bearing mice. Tumor growth was monitored with luminescence activity on a weekly basis from the day of MOSEC/luc cells challenge.

### Statistical analysis

All data expressed as mean ± SD are representative of at least two different experiments. Comparisons between individual data points were made using a Student's *t *test. Differences in survival between experimental groups were analyzed using the Kaplan-Meier approach. The statistical significance of group differences will be assessed using the log-rank test.

## Results

### Doxorubicin is taken up by MOSEC tumor cells leading to tumor cell death

**Figure 1:**
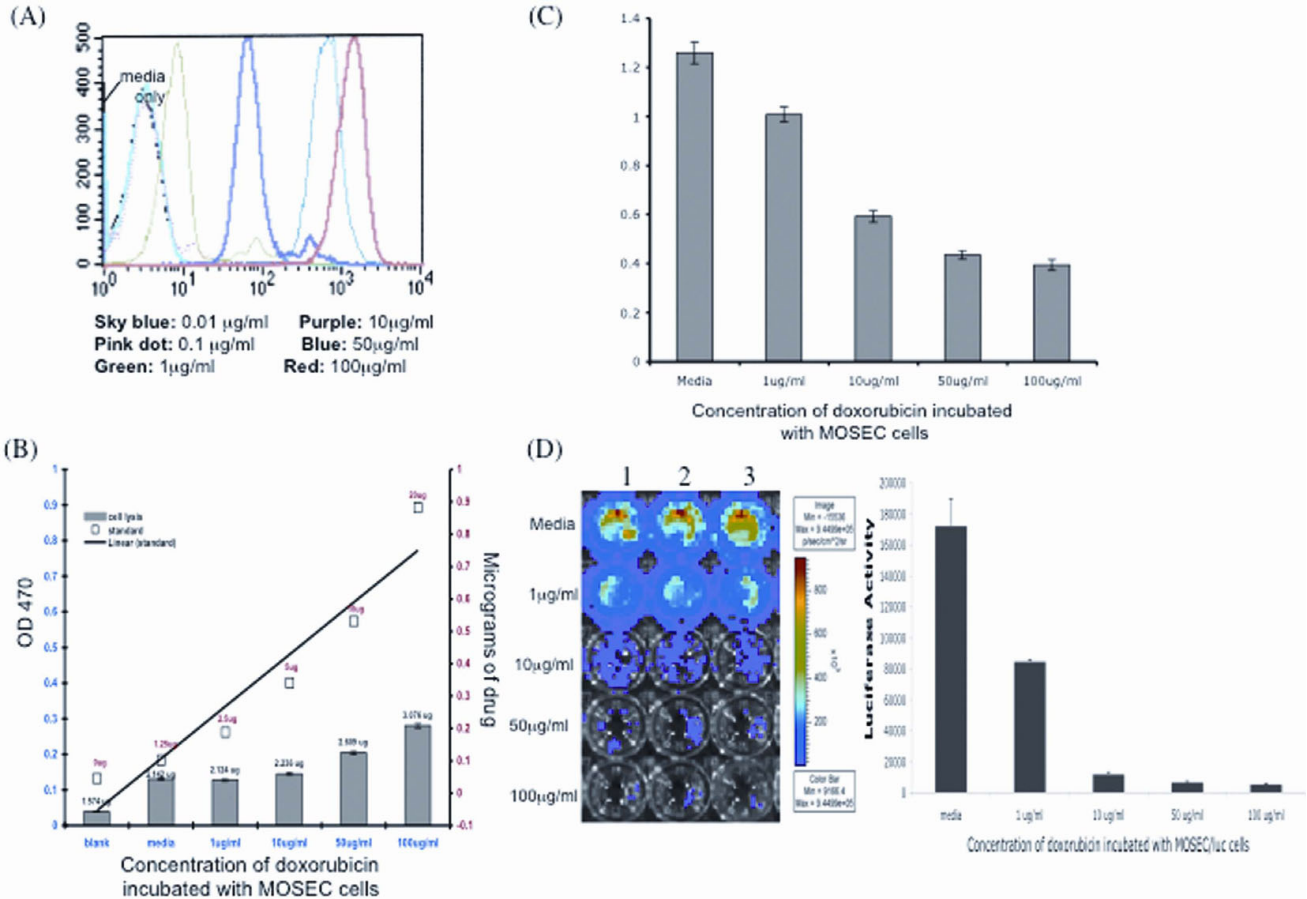
**Characterization of doxorubicin treatment of tumor cells**. MOSEC or MOSEC/luc tumor cells (1 × 10^6^) were cultured in the presence of different doses of doxorubicin: 0, 0.01, 0.1, 1,10, 50, 100 μg/ml. Flow cytometry was performed on doxorubicin-treated MOSEC cells (MOSEC-dox) at 2 hrs of incubation. **(A) **Flow cytometry showing uptake of doxorubicin at each concentration by the MOSEC tumor cells. Another pool of MOSEC cells incubated for 2 hrs with doxorubicin were spun to form cell-pellets, which were lysed with protein-extraction buffer and added to DMSO. The amount of doxorubicin in the cell lysate solution was determined using spectrophotometry along with generating a standard curve. **(B) **Bar graph superimposed under standard curve showing the amount of doxorubicin inside the MOSEC cells for each concentration after extraction from cell lysates. The numbers above each bar indicate μg of doxorubicin per 1 × 10^6 ^cells. The left *y*-axis indicates optical density reading at 470 nm; the right *y*-axis indicates micrograms of doxorubicin used to generate the standard curve. A pool of MOSEC tumor cells was incubated with doxorubicin for 24 hrs and an MTT assay was then performed. **(C) **Representative bar graph from the MTT data showing the viability of MOSEC cells after incubation with different concentrations of doxorubicin. MOSEC/luc tumor cells which were incubated with doxorubicin for 24 hrs were imaged using bioluminescence IVIS systems. **(D) **Luminescence image showing luciferase activity in viable MOSEC/luc cells. The numbers at the top indicate 3 identical trials of the same experiment. The bar graph depicts the kinetic expression of luciferase in MOSEC/luc cells incubated with different amounts of doxorubicin.

To characterize whether doxorubicin can be taken up by tumor cells and lead to tumor cell death, we performed various *in vitro *experiments using MOSEC and luciferase-expressing MOSEC (MOSEC/luc) tumor cells. Pools of MOSEC and MOSEC/luc cells (1 × 10^6^) were incubated with different concentrations of doxorubicin. Since an intrinsic characteristic of doxorubicin is auto-fluoresence, after 2 hours of incubation with the drug, the doxorubicin-treated MOSEC cells were subjected to flow cytometry analysis. As shown in Figure 1A, histograms of the doxorubicin-treated cell populations demonstrated increased shift with increasing concentrations of administered drug. We then checked the amount of doxorubicin taken up by the MOSEC tumor cells. After 2 hours of incubation with the drug, pools of differing concentrations of doxorubicin -treated MOSEC cells were collected and spun down to form cell pellets. Protein-extraction buffer was added to lyse the cells and release the intracellular doxorubicin and the amount of drug inside the different cell pools was determined by a standard curve using spectrophotometry analysis. We found that the amount of intracellular doxorubicin for each cell population increased with increasing concentrations of doxorubicin placed in the media, as shown in Figure 1B. The rest of the drug remains in the solution. To check the effects of doxorubicin on the viability of MOSEC cells, the tumor cells were incubated with doxorubicin for 24 hours. We then performed MTT assays to determine the viability of tumor cells after exposure to doxorubicin. Figure 1C illustrates that with increasing concentrations of doxorubicin, the numbers of viable MOSEC cells available to convert MTT decreases, resulting in decreasing OD values. We also checked the effects of doxorubicin on MOSEC/luc cells. After 24 hours of incubation, luciferin was added to doxorubicin-treated MOSEC/luc cells followed by bioluminesence imaging. As shown in Figure 1D, the luciferase activity in viable MOSEC/luc cells decreased with increasing concentrations of doxorubicin. Thus, our data suggests that incubation of doxorubicin with MOSEC and MOSEC/luc tumor cells leads to intracellular drug uptake by the tumor cells, subsequently leading to cell death. Furthermore, the degree of tumor cell death induced by the drug increases with increasing concentration of doxorubicin.

### Transfer of doxorubicin from doxorubicin-loaded MOSEC cells to untreated MOSEC cells (MOSEC-GFP) is mediated through cells being in close vicinity of each other

**Figure 2:**
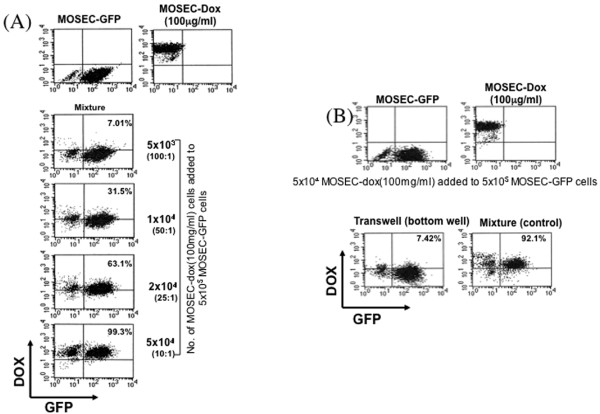
**Flow cytometry analysis of MOSEC-GFP tumor cells incubated with MOSEC-dox cells mixed together or separated by a transwell membrane**. MOSEC cells incubated for 2 hrs in with doxorubicin (MOSEC-dox) at a concentration of 100 μg/ml were added to MOSEC-GFP cells (5 × 10^5^/well) in various amounts according to the indicated ratios. Another pool of MOSEC-dox cells (5 × 10^4^) were added to the upper plate of a transwell system with 5 × 10^5 ^MOSEC-GFP cells in the bottom plate. A transwell system in which the MOSEC-dox cells were mixed together with MOSEC-GFP cells was used as a control. At 24 hrs of culture, the cells from the mixtures and from the bottom plate of the transwell system were collected and analyzed for presence of GFP and doxorubicin using flow cytometry. Representative figures from the flow cytometry data of **(A) **the different mixtures of MOSEC-GFP cells incubated with differing amounts MOSEC-dox cells and **(B) **bottom well containing MOSEC-GFP cells of the transwell system, with data from the control mixture to the right. The numbers in the upper right hand corner show the percentage of total collected cells that indicate presence of GFP and doxorubicin.

One of the serious side effects of doxorubicin as a chemotherapeutic agent is cardiotoxicity. Therefore, targeted delivery of the chemotherapeutic drug to tumor cells can potentially reduce the dose required for the treatment and the systemic toxicity of the drug. In order to test whether the doxorubicin in drug-loaded MOSEC tumor cells could be transferred to other MOSEC tumor cells, we performed flow cytometry experiments. We found a certain number of MOSEC cells expressed GFP *and *demonstrated red fluorescence of doxorubicin. Figure 2A shows that the percentages of the double positive cells from the total collected cells increased with increasing ratios of added MOSEC-dox cells. This suggests that doxorubicin was transferred from the MOSEC-dox cells to the MOSEC-GFP cells. To determine if the drug transfer requires the cells to be contact or in close vicinity of each other, we performed another co-culture experiment utilizing a transwell system to physically separate the cells during incubation. MOSEC-GFP cells were plated in the bottom well and MOSEC-dox cells were added to upper well. After 24 hours, we evaluated the percentage of cells collected from the bottom well of the transwell system that showed presence of GFP and red fluorescent doxorubicin. We found that a significantly lower percentage of cells collected from the transwell showed presence of GFP and doxorubicin, as shown in Figure 2B, compared to the experimental control. These results support that cell-to-cell contact or presence of cells in close vicinity of each other is required for doxorubicin drug transfer from MOSEC-dox cells to MOSEC-GFP cells.

**Figure 3:**
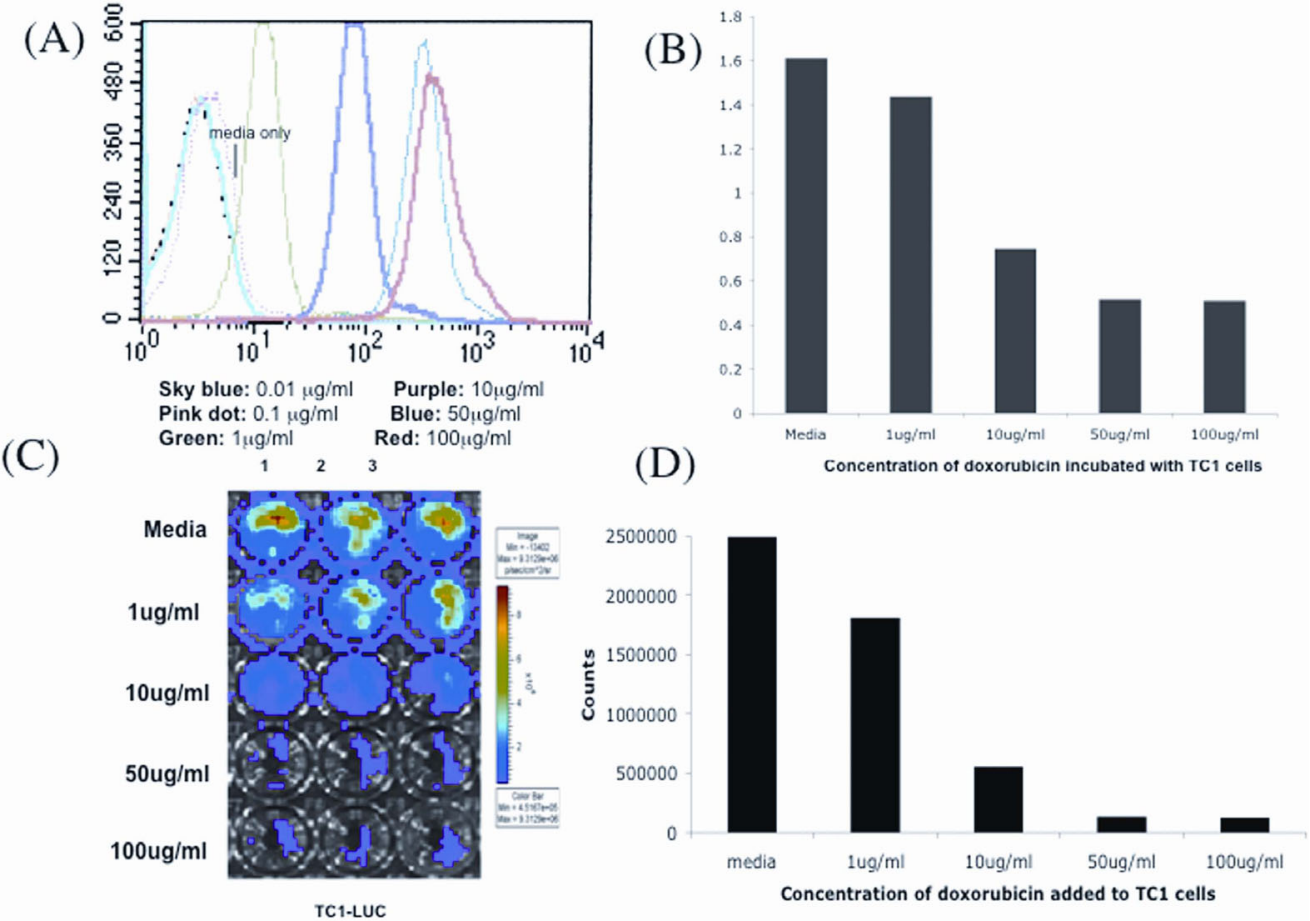
**Characterization of doxorubicin-treated TC1 cells**. TC1 or TC1/luc tumor cells (1 × 10^6^) were cultured in the presence of different doses of doxorubicin: 0, 0.01, 0.1, 1, 10, 50, 100 μg/ml. Flow cytometry was performed on doxorubicin-treated TC1 cells at 2 hrs of incubation. An MTT assay was performed with another pool of TC1 tumor cells incubated for 24 hrs with doxorubicin. Bioluminescence imaging was done with TC1/luc tumor cells incubated with doxorubicin for 24 hrs after adding luciferin. **(A) **Flow cytometry showing uptake of doxorubicin at each concentration by the TC1 tumor cells. **(B) **Representative bar graph from the MTT data showing the viability of TC1 cells after treatment with different concentrations of doxorubicin. (C) Bioluminescence image showing luciferase activity in TC-1 cells remaining after incubation with doxorubicin. (D) Bar graph depicting the kinetic expression of luciferase in TC-1 cells incubated with different amounts of doxorubicin.

**Figure 4:**
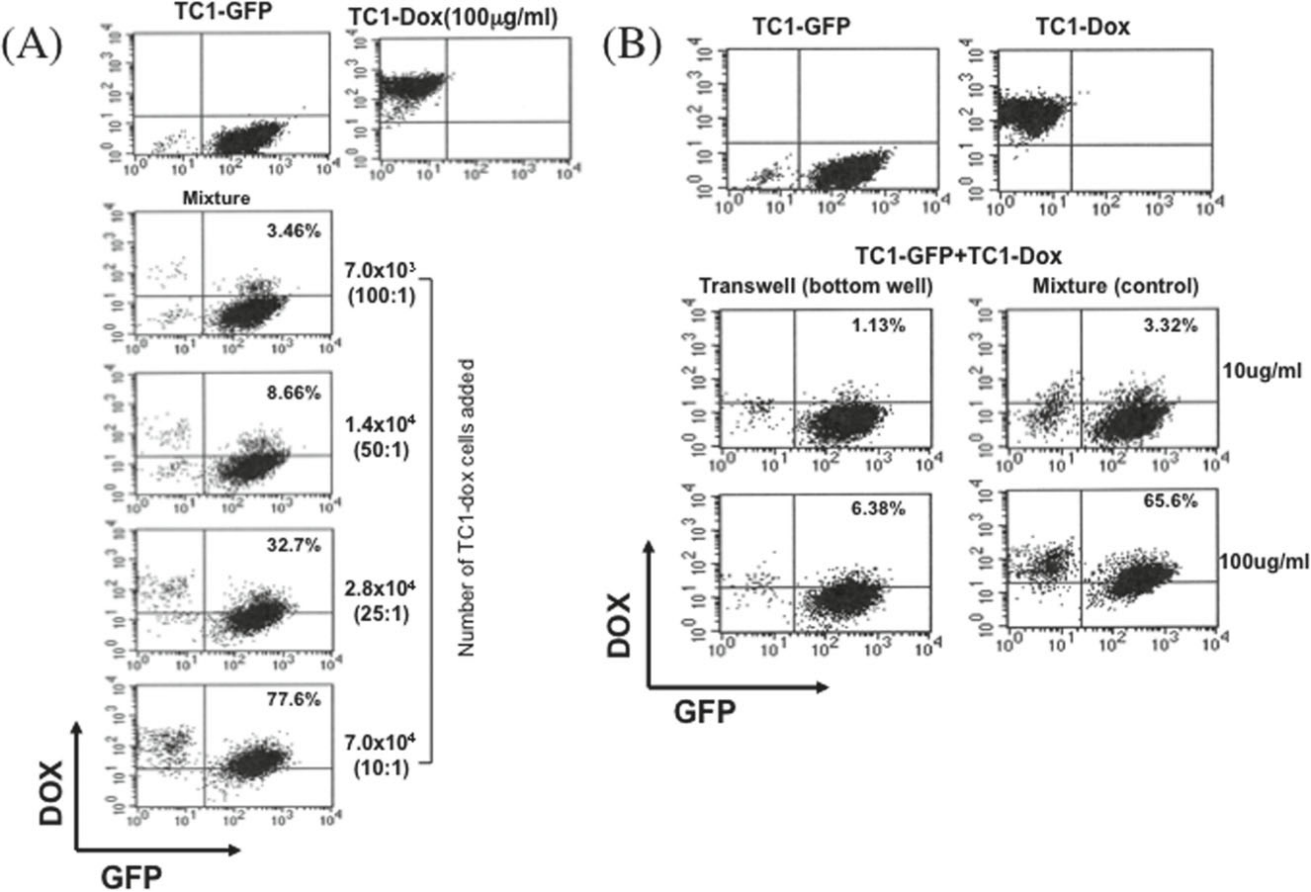
**Flow cytometry analysis of TC1-GFP tumor cells incubated with doxorubicin-treated TC1 cells mixed together or separated by a transwell membrane**. TC1 cells incubated for 2 hrs in the presence of doxorubicin (100 μg/ml) were added to TC1-GFP cells (7 × 10^5^/well) in various amounts according to ratios indicated. Doxorubicin (10 or 100 μg/ml)-treated TC1 cells (7 × 10^5^) were also added to the upper plate of a transwell system with the TC1-GFP cells (7 × 10^4^) in the bottom plate. A control for the transwell experiment in which the doxorubicin-treated TC1 cells were again mixed with TC1-GFP cells was done. At 24 hrs of culture, the cells from the mixture and from the bottom plate of the transwell system were collected and analyzed using flow cytometery. (A and B) Representative flow cytometery data of **(A) **TC1-GFP cells mixed with differing amounts of doxorubicin-treated TC1 cells and **(B) **TC1-GFP cells from the bottom well of the transwell system, the control data to the right. The numbers in the upper right hand corner of each dot plot show the percentage of cells that contain GFP and doxorubicin.

We also performed the same *in vitro *experiments using TC-1 and TC-1/luc cells. In the TC-1 cell line, we found similar results to what we found in the MOSEC cell line. TC-1 cells took up doxorubicin after 2 hours of incubation and were killed after 24 hours (Figure 3). Furthermore, we found that transfer of doxorubicin from TC-1-dox cells to TC-1/luc cells required cell-to cell contact or presence of cells in close vicinity of each other (Figure 4).

### MOSEC-luc cells incubated with MOSEC-dox cells are killed via transfer of doxorubicin

**Figure 5:**
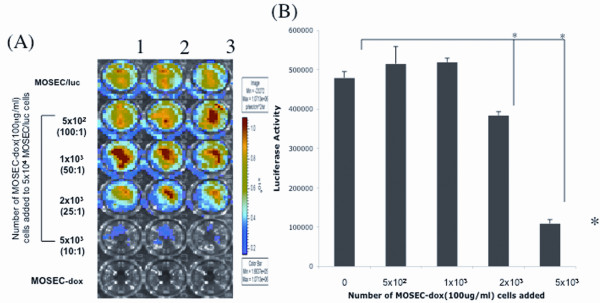
***In vitro *tumor killing assay**. MOSEC/luc tumor cells (5 × 10^4^/well) were plated in 24-well plates. Different amounts of MOSEC-dox(100 μg/ml) doxorubicin were added to the MOSEC/luc cells according to the indicated ratios. After 48 hrs, luciferin was added to the cells 7-8 min before bioluminescence images were taken of the cells. **(A) **Representative bioluminescent image of the tumor cells at 2 days of incubation. The numbers at the top indicate 3 identical trials of the same experiment. **(B) **Bar graph depicting the measured expression of luciferase in the viable MOSEC/luc tumor cells. *p < 0.01.

In order to determine whether the transfer of doxorubicin would have a cytotoxic effect on target cells, we performed *in vitro *tumor killing assays using luciferase-expressing MOSEC tumor cells (MOSEC/luc). MOSEC-luc cells were plated in 24-well plates and increasing amounts of MOSEC-dox cells were added according to fixed ratios. We found that MOSEC/luc cells were killed after incubation with MOSEC-dox cells through the direct transfer of doxorubicin. As shown in Figure 5A, the luminescent intensity in the MOSEC/luc cells decreases with increasing numbers of added MOSEC-dox cells. The bioluminescence is an indirect measure of viability of the tumor cells that can exhibit luciferase activity. The bar graph in Figure 5B illustrates the decreasing levels of luminescent intensity as the numbers of MOSEC-dox cells increase. Thus, our data suggests that after 48 hours of incubation, doxorubicin-treated MOSEC cells can cause cell death among MOSEC/luc cells. Furthermore, higher numbers of MOSEC-dox cells incur greater levels of tumor cell killing.

### Doxorubicin is transferred from MOSEC-dox cells to MOSEC-GFP cells *in vivo*

**Figure 6:**
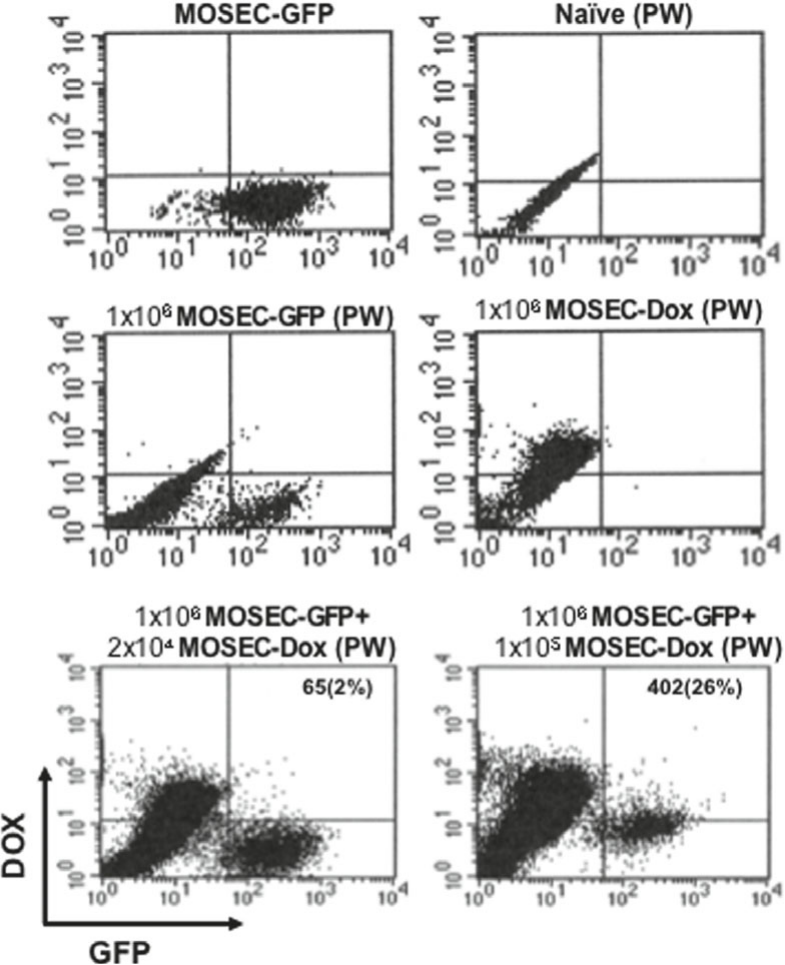
**Flow cytometry analysis of peritoneal cells after intraperitoneal injections of MOSEC-GFP and MOSEC-dox cells**. MOSEC-GFP tumor cells (1 × 10^6^/mouse) were intraperitoneally injected into groups of C57BL/6 mice, followed 1 day later by intraperitoneal injection of MOSEC-dox cells (2 × 10^4 ^or 1 × 10^5^/mouse). As controls, C57BL/6 mice were injected with only MOSEC-GFP or MOSEC-dox cells or no cells. One day after the last injection, all mice were sacrificed. Cells were collected from the intraperitoneal cavity by peritoneal wash (PW) and analyzed using flow cytometry specific for GFP and doxorubicin. Representative figures from the flow cytometry data showing migration of peritoneal wash cells collected from the mice injected with MOSEC-GFP and MOSEC-dox cells.

In order to determine whether the transfer of doxorubicin by drug-loaded tumor cells seen in cell culture would also occur *in vivo*, we inoculated C57BL/6 mice with MOSEC-GFP tumor cells intraperitoneally. We then injected the mice one day later with different amounts of MOSEC-dox cells according to fixed ratios. 24 hours later, the intraperitoneal cells were collected from the peritoneal cavity and analyzed using flow cytometry for the presence of intracellular doxorubicin and GFP. We found that transfer of doxorubicin between cells also occurred *in vivo*. As shown in Figure 6, a subset of collected intraperitoneal cells both expressed GFP and showed red fluorescence by doxorubicin. The percentages of cells that contained both doxorubicin and GFP increased with higher numbers of added MOSEC-dox cells. Overall, the percentages of total collected cells that showed presence of doxorubicin and GFP from the *in vivo *experiment were lower than the percentages from the *in vitro *experiments. This can be explained by the number of endogenous, non-cancerous, intraperitoneal cells collected and evaluated as part of the total number of intraperitoneal cells assayed. Thus, our data suggests that transfer of doxorubicin observed *in vitro *between MOSEC-dox and MOSEC-GFP cells can also occur *in vivo*.

### Administration of irradiated MOSEC-dox tumor cells to MOSEC/luc tumor-bearing mice leads to decreased tumor burden

**Figure 7:**
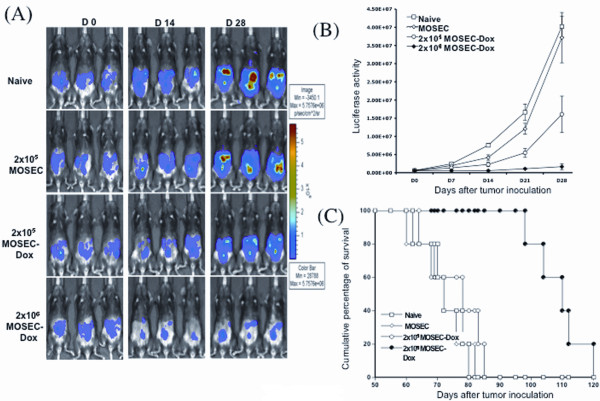
***In vivo *tumor treatment experiment**. C57BL/6 mice were inoculated with 5 × 10^5^/mouse of MOSEC/luc tumor cells. Four days later, MOSEC/luc tumor-bearing mice were treated with a low (2 × 10^5^/mouse) or high (2 × 10^6^/mouse) numbers of irradiated MOSEC-dox(100 μg/ml) tumor cells. As controls, groups of MOSEC/luc tumor-bearing mice were treated with irradiated MOSEC cells (2 × 10^5^/mouse) or left without treatment (naïve). Survival analysis was also performed of the different groups of mice. **(A) **Representative bioluminescence images of MOSEC/luc tumor-bearing mice treated with the different numbers of doxorubicin-treated MOSEC tumor cells. **(B) **Line graph illustrating the measured values of luminescent intensity in the different groups of mice. **(C) **Kaplan-Meier survival analysis of MOSEC/luc tumor-bearing mice treated with low and high numbers of doxorubicin-treated MOSEC cells compared to the control groups.

We examined the doxorubicin-treated MOSEC cells as a modality of treatment for MOSEC tumors. C57BL/6 mice were inoculated with MOSEC/luc cells. After 4 days, groups of tumor-bearing mice were administered either different doses of irradiated MOSEC-dox cells. One group of tumor-bearing mice administered irradiated MOSEC cells or no treatment were used as controls. Luminescence activity has been shown to correlate well with tumor load using luciferase-expressing tumor cells in previous studies by us and other groups, [15-18]. Thus, we believe luciferase activity can be used as a suitable indicator of tumor load in tumor-bearing mice. As shown in Figure 7A, the size of tumors as indicated by the levels of luciferase activity were decreased in tumor-bearing mice treated with higher number of irradiated MOSEC-dox cells. The luciferase activity was quantified as illustrated in Figure 7B. This indicates that the administration of MOSEC-dox cells to MOSEC/luc tumor-bearing mice led to significantly decreased tumor growth. Tumor-bearing mice treated with MOSEC-dox cells/mouse also showed improved survival compared to the other groups (Figure 7C). Thus, our data suggests that higher numbers of irradiated MOSEC-dox cells can be used to treat MOSEC/luc tumor bearing C57BL/6 mice and can improve survival.

### Administration of irradiated, pre-treated MOSEC cells with high levels of doxorubicin to MOSEC/luc tumor-bearing athymic nude mice leads to decreased tumor burden

**Figure 8:**
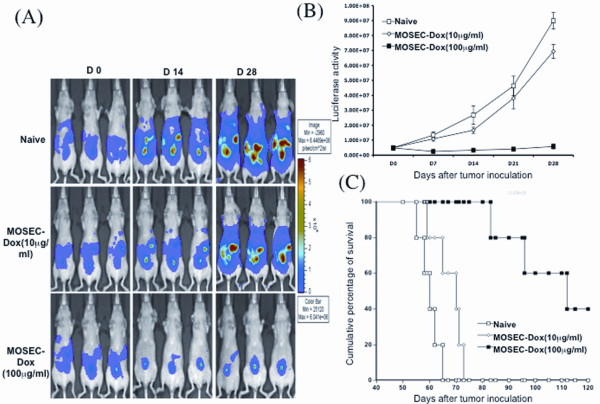
**Characterization of the anti-tumor effects of MOSEC-dox tumor cells in nude mice**. Athymic nude mice were inoculated with 2.5 × 10^5^/mouse of MOSEC/luc tumor cells. Four days later, MOSEC/luc tumor-bearing mice were treated with 2 × 10^6^/mouse of irradiated MOSEC-dox(10 μg/ml or 100 μg/ml) tumor cells. A group of MOSEC/luc tumor-bearing nude mice without treatment was used as a control (naïve). Survival analysis was also performed of the different groups of mice. **(A) **Representative bioluminescence images of different MOSEC-dox treated mice compared to the control. **(B) **Line graph illustrating the kinetic expression of luciferase in the different MOSEC-dox treated mice compared to the control. **(C) **Kaplan-Meier survival analysis of different MOSEC-dox treated mice compared to the control.

We also examined irradiated MOSEC-dox cell vaccination as treatment in athymic nude tumor-bearing mice, which would allow us to characterize the antitumor effect without involvement of T cell-mediated immune responses. Mice were inoculated with MOSEC/luc cells. After four days, groups of tumor-bearing nude mice were administered irradiated MOSEC cells pre-treated with doxorubicin. We found that administration of irradiated MOSEC-dox cells that were pre-treated with 100 μg/ml of doxorubicin to MOSEC/luc tumor-bearing mice led to significantly decreased tumor growth. Administration of irradiated MOSEC-dox cells that were pre-treated with 10 μg/ml of doxorubicin had little to no effect on tumor growth. As shown in Figure 8A, the sizes of tumors as indicated by the levels of luciferase activity are decreased in tumor-bearing mice treated with the irradiated MOSEC-dox cells incubated with the higher concentration of doxorubicin. The luciferase activity was quantified as illustrated in Figure 8B. Furthermore, tumor-bearing nude mice treated with the irradiated MOSEC-dox(100 μg/ml) cells showed enhanced survival compared to the other mice groups (Figure 8C). Thus, our data suggests that irradiated MOSEC-dox cells that have been incubated with a high concentration of doxorubicin can be used to treat MOSEC/luc tumors in athymic nude mice, leading to significantly reduced tumor burden and prolonged survival.

### Irradiated MOSEC-dox tumor cells are more effective than doxorubicin alone as treatment for MOSEC/luc tumors

**Figure 9:**
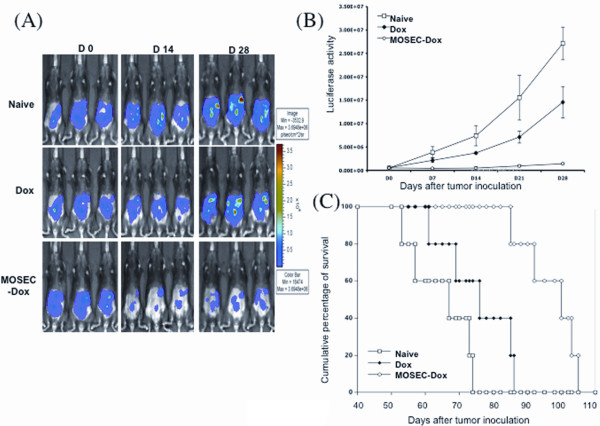
**Comparison of different doxorubicin-based treatments**. C57BL/6 mice were inoculated with 5 × 10^5^/mouse of MOSEC/luc tumor cells. Four days later, MOSEC/luc tumor-bearing mice were treated with 2 × 10^6^/mouse of doxorubicin (100 μg/ml)-treated, irradiated MOSEC tumor cells. The concentration of doxorubicin inside doxorubicin-treated MOSEC cells was determined as described in the Materials and Methods. Comparable amount of doxorubicin to that found collectively inside 2 × 10^6 ^of doxorubicin (100 μg/ml)-treated MOSEC cells was set as 0.5 mg/kg (10 μg/mouse). Another group MOSEC/luc tumor-bearing mice were treated intraperitoneally with 0.5 mg/kg of doxorubicin alone for comparison. A group of MOSEC/luc tumor-bearing mice without treatment was used as a control (naïve). Survival analysis of the different mice groups was also performed. **(A) **Representative bioluminescence images of MOSEC/luc tumor-bearing mice treated with the different doxorubicin-based treatments. **(B) **Line graph illustrating the measured values of luminescent intensity in the different groups of mice. **(C) **Kaplan-Meier survival analysis of MOSEC/luc tumor-bearing mice treated with the doxorubicin-based treatments compared to the control groups.

We compared doxorubicin-treated MOSEC cells to doxorubicin alone as treatment for MOSEC tumors. C57BL/6 mice were inoculated with MOSEC/luc cells. After 4 days, one group of tumor-bearing mice was administered irradiated MOSEC-dox cells pre-treated with 100 μg/ml of doxorubicin. Based on Figure 1B, we determined the concentration of intracellular doxorubicin in the MOSEC cells pre-treated with 100 μg/ml of doxorubicin. For comparison, another group of tumor-bearing mice was administered doxorubicin alone. We found that administration of irradiated MOSEC-dox cells to MOSEC/luc tumor-bearing mice led to decreased tumor growth; whereas, administration of 10 μg of doxorubicin alone to MOSEC/luc tumor-bearing mice led to little or no antitumor effects. As shown in Figure 9A, the size of tumors as indicated by the levels of luciferase activity are decreased in tumor-bearing mice treated with the irradiated MOSEC-dox cells. Treatment of tumor-bearing mice with a comparable level of doxorubicin did not lead to a significant decrease in tumor sizes. The luciferase activity was quantified as illustrated in Figure 9B. Furthermore, tumor-bearing mice treated with irradiated MOSEC-dox cells showed enhanced survival compared to the other groups (Figure 9C). Thus, our data suggests that delivery of small amounts of drug via irradiated tumor cells containing doxorubicin is more effective in treating MOSEC/luc tumor-bearing mice than direct treatment with the drug alone.

## Discussion

In the current study, we generated a chemotherapeutic drug delivery system using irradiated MOSEC tumor cells which was capable of delivering the drug to other MOSEC tumor cells in tumor-bearing mice to result in potent therapeutic antitumor effects. Using the unique property of doxorubicin's red auto-fluoresence, we found that incubation of MOSEC cells with doxorubicin led to the intracellular uptake of the drug and the eventual death of the tumor cells. We also found that drug-loaded tumor cells were capable of transferring the drug to other non-drug-loaded tumor cells in close vicinity. In addition, we found that the use of irradiated MOSEC-dox cells to deliver doxorubicin is more effective in treating MOSEC/luc tumors than administration of a comparable dose of doxorubicin alone. Thus, our study suggests that local delivery of chemotherapeutic drugs by tumor cells may require significantly less amount of drug to control ovarian cancer. The success of the current study warrants further exploration of such a delivery approach using other chemotherapeutic drugs for the treatment of cancers.

Our study shows that irradiated tumor cells loaded with a chemotherapeutic drug can lead to the control of MOSEC tumors. We have revealed that this delivery system is capable of transferring doxorubicin to other tumor cells *in vitro *and *in vivo *resulting in tumor cell death. The mechanism of chemotherapeutic action of doxorubicin on cancer cells is through DNA intercalation and topoisomerase II enzyme inhibition [19]. Through these two actions, doxorubicin can disrupt cellular processes involving DNA such as synthesis and transcription, leading to cell death. Thus, we can reason that the antitumor effects observed as a result of treatment with irradiated MOSEC-dox tumor cells can be partly attributed to doxorubicin-mediated tumor-cell killing. Other contributing factors for the observed therapeutic effects include chemotherapy-induced cell death and subsequent antitumor activity based on activation of the immune system. Our previous studies have shown that tumor cells treated with chemotherapy can lead to tumor cell death, resulting in activation of tumor-specific immunity [20-22].

The observed antitumor effects generated by doxorubicin-loaded tumor cells may also be contributed by tumor-specific immunity. Recent studies have shown that anthracycline drugs including doxorubicin induce the rapid, preapoptotic translocation of calreticulin (CRT) to the cell surface and result in improved processing of tumor cells by dendritic cells [23]. Thus, the expression of CRT on the surface of tumor cells mediated by doxorubicin may play an important role in the generation of anticancer immune responses. Thus, doxorubicin-loaded tumor cells may generate antitumor effects through doxorubicin-mediated killing as well as tumor-specific immunity.

It is important to consider issues related to safety and feasibility for the use of this novel delivery system in clinics. While this delivery system is used to deliver the drug directly to tumor cells, it is possible that MOSEC-dox cells may also deliver doxorubicin to healthy fibroblasts and other cells. This raises concerns for toxicity. Nevertheless, we expect that normal cells such as fibroblasts would be less susceptible to the effects of the drug as compared to tumor cells at the same concentration of drug delivered by MOSEC-dox. This is generally true in the case of free form of doxorubicin. In addition, intraperitoneal mode of delivery of irradiated tumor cells loaded with drug would potentially have less systemic toxicity compared to intravenous drug delivery. The irradiation of drug-loaded tumor cells will further alleviate concerns for growth of the drug-loaded tumor cells following injection. Furthermore, the principle generated from the current study provides the rationale for further exploration of alternative options for drug delivery such as controlled release biodegradable polymers [24,25] or non neoplastic cells from patients such as fibroblasts or PBMCs. It will be important to further test whether these kinds of reagents will be able to generate equivalent or better effects compared to the current approach.

In summary, our study demonstrates that the employment of drug-loaded irradiated tumor cells represents a potentially innovative approach for the delivery of chemotherapeutic drugs for the control of ovarian tumors. Further exploration in this area will create the opportunity for the development of innovative chemotherapy regimens for the control of ovarian cancer.

## Competing interests

The authors declare that they have no competing interests.

## Authors' contributions

DK was involved in the execution of the project. TH and AM were involved in the interpretation of the data and writing the manuscript. AW and WTH performed some of the experiments. SIP and CFH provided overall supervision and guidance for the project. All authors read and approved the final manuscript.
